# Environmental factors controlling potentially toxic element behaviour in urban soils, El Tebbin, Egypt

**DOI:** 10.1007/s10661-019-7388-1

**Published:** 2019-04-06

**Authors:** Ibrahim Said, Salman Abd El-Raof Salman, Yousria Samy, Samir Ahmed Awad, Ahmed Melegy, Andrew S. Hursthouse

**Affiliations:** 1 Department of Geoscience, National Research Centre, Dokki, Giza, Egypt; 2 Department of Geology, Faculty of Science, Ain Shams University Abbasya, Cairo, Egypt; 3 School of Computing, Engineering and Physical Sciences, University of the West of Scotland, Paisley, PA1 2BE UK

**Keywords:** Potentially toxic element, Urban soil, Helwan, Egypt, Sequential chemical extraction, Bioavailability, Multivariate statistical analysis

## Abstract

This study focuses on the assessment of surface soils from industrially polluted region (El Tebbin) of southern Cairo, Egypt. The impact of agricultural, residential and industrial land use on soils developed from Nile river sediments has significantly compromised their function. Previous evidence has shown that the food chain is contaminated and enhances risk of contaminant exposure of the residential communities. This study investigates factors controlling potentially toxic element (PTE) distribution (Co, Ni, Pb, Cd, Zn, Cr and Cu) in El Tebbin soils and provide estimates of their mobility and bioavailability. The PTE concentrations are characterised by high variability as result of the variety of natural and anthropogenic influences. Highest spatial variability is found for Zn, Cd, Pb and Cu (C.V = 260.0%, 280.4%, 140.8% and 159.6% respectively) and enrichment factors indicate strong anthropogenic inputs. For Co and Ni, relatively low spatial variability (C.V = 65.8% and 45.0% respectively) with depletion in Ni suggests a relatively minor contribution from anthropogenic sources. For Cr, a more uniform distribution pattern showing depletion to minimal enrichment across the study area (C.V = 19.2%) reflects almost exclusive lithogenic control. Using principle component analysis (PCA) to explore concentration data reveals that the major inputs affecting PTE distribution are modified by primary soil properties (texture and pH). Their relative bioavailability (identified through sequential chemical extraction) relates strongly to local input sources. Those elements dominated by lithogenic input (Ni and Co) were found predominantly in soil residual fractions (95.6% and 90.5% respectively), while elements with stronger anthropogenic contributions (Cd, Zn, Pb and Cu) showed much higher portion in the more mobile and bioavailable fractions obtained from sequential chemical extraction, with average proportions of the totals being 62.6%, 57%, 40.7% and 39.2% respectively. Those PTEs with strong anthropogenic influence are potentially much more mobile for bioaccumulation in food chain with increased health risk for exposed residents and are confirmed by elevated concentrations of Cd, Zn, Pb and Cu recorded in local plant species. The main pollution sources were further highlighted by cluster analysis and showed vehicle traffic and specific industrial activities but which varied significantly from site to site. The identification of sources through the approach developed here allows prioritisation of monitoring and regulatory decisions by the local government to reduce further environmental exposure of the local population.

## Introduction

It is widely accepted that environmental contamination with potentially toxic elements (PTE) is one of the largest threats to human health. For example, Pb accumulates the human body causing damage to brain and nervous system (Edwin 2011). The liver and kidney are the major target organs of Cd accumulation. However, some PTEs are essential for biota life in traces but in excess may become toxic. The elements Cu, Co and Zn are essential for growth and their deficiency causes biological deterioration; they can also have toxic effects at higher concentrations (Kabata-Pendias 2010). They reach terrestrial ecosystems from natural and anthropogenic sources. The natural sources are parent rock weathering or weathering and transport of soils and sediments in the catchments. The anthropogenic factors are traffic emission (vehicle exhaust particles, tire wear particles, brake lining wear particles), industrial, domestic emission, mining, pesticides, fertilisers and atmospheric deposition ((Montagne et al. 2007); (Morton-Bermea et al. 2009) and (Elnazer et al. 2015).

Environmental impact of a PTE depends on its geochemical association in the soil which is a function of the origin of the metal and its reactivity under current or changing environmental conditions. Different geochemical associations of PTE have different mobility and the potential effect of the pollution is either enhanced or retarded thorough this association and local conditions. Operationally defined (chemical) availability is a widely used tool to provide indicators of likely biological availability and wider environmental risk. A number of extraction protocols are commonly used which can identify potential environmental impact for PTE associations in each fraction (Bacon and Davidson 2008). The principle of the approach being that the expected availability/mobility generally reduces with increasing severity of extraction conditions, with a notional association with increasingly stable solid phases. This depends very much on the nature of the soil medium—input parent materials, weathering and any disturbance or addition of anthropogenic materials and for surface soils makes it very spatially and temporarily variable. The extraction scheme developed in the late 1970s (Tessier et al. 1979) is still one of the most popular and its modification and its application to a wider range of environmental solid phases continues to be the subject of discussion (Rodgers et al. 2015). Reactivity and predicted environmental availability declines in the order of increasing reagent aggression which are often associated with notional mineralogical phases ((Harrison et al. 1981); (Bacon and Davidson 2008)). The sequence is exchangeable (F1) (magnesium chloride) > carbonate (F2) (acetic acid) > Fe/Mn oxides (F3) (hydroxylammonium hydrochloride) > organic (F4) (acidified hydrogen peroxide) > residual (F5) (strong acid). The mobility of elements decreases in the order of the extraction sequence.

Exchangeable phase (F1) is readily available for plant uptake, and other fractions depend on several mobilisation/immobilisation processes (Förstner 1989). Elements bound predominantly to carbonate and organic fractions will be easily mobilised to the environment under acidic and oxidative conditions while these bound to oxides would be released into the environment under reducing conditions. Elements associated with natural (silicate mineral) parent materials occur primarily in the residual fraction (F5) and have low biological availability which reduces their toxic risk. Elements associated with the residual fraction (F5) are mainly incorporated in the mineral structure. Therefore, extraction of them from the residual fraction (F5) requires the application of aggressive extracting solution. We outline a study of the assessment of PTE availability in relation to land use and the significance of the association in relation to soil factors and spatial distribution.

**Fig. 1:**
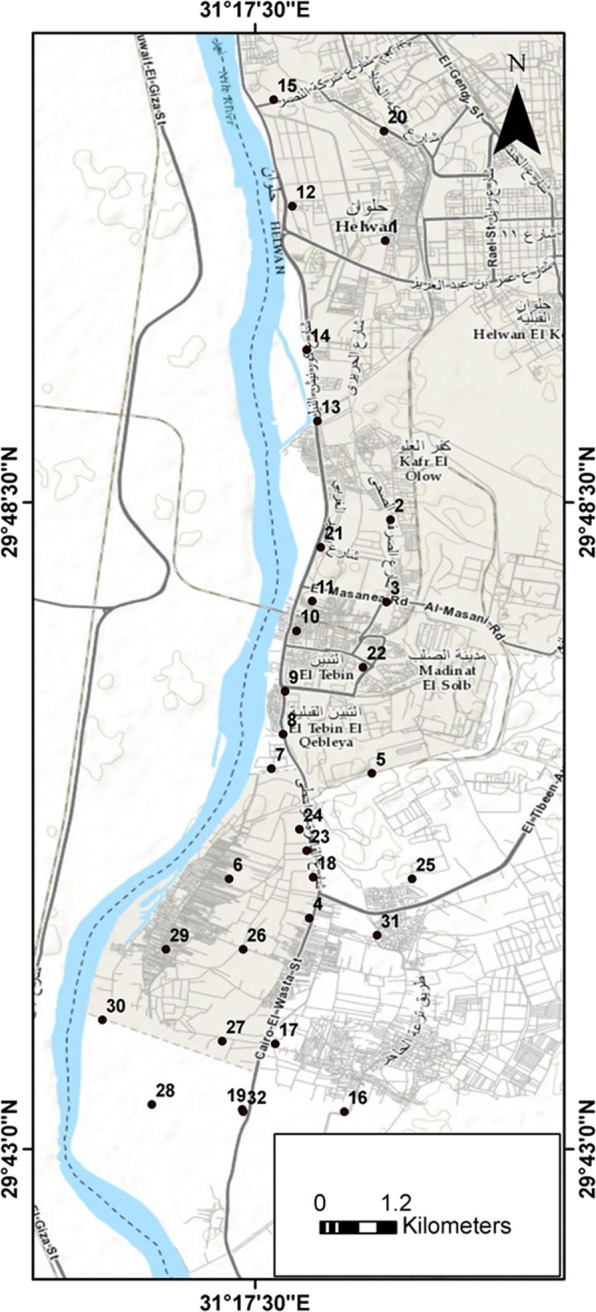
Map of the study area showing soil sampling sites (land use classification summarised in Table 1)

Egypt shares many of the environmental problems of developing and emerging countries. The oldest and biggest industries in Egypt are located in the historical Helwan district in the southern suburb of Cairo on the eastern bank of the river Nile. This region was developed as a major industrial zone during the 1950s and 1960s, merging into a suburb of Cairo. The dominant industrial activities include cement plants, electrical power stations, iron and steel industry, nonferrous metallurgical work, coke, car manufacturing and a number of other minor pollution sources (El-Mekawy 2007). The district of Helwan can be divided into three regions; with domestic, industrial and agricultural farms (producing small scale fruit, vegetables, grazing of livestock). It is located about 20 km south of Cairo and extends between longitudes 31^°^ 16’–31^°^ 21’ E and latitudes 29^°^ 43’–29^°^ 52’ N (Fig. 1). The study area covers about 94.7 km^2^. The high concentration of industrial activity coupled with high average temperatures (monthly average = 13.8 °C min; 28.7 °C max), lack of rain (monthly average 5 mm (max); 0 mm (min)) and predominantly low wind speed (Climate-Data 2018) favour the accumulation of air pollutants over the district and the formation of air pollution episodes is recognised in routine monitoring (EEAA 2018). The situation temporarily worsens with dust-laden winds especially during spring and autumn. The agricultural soil in the Nile valley originates from the weathering of the mafic/ultramafic rocks of the Ethiopian plateau and deposition as sediment downstream. The thickness of this flood plain soil is about 1.5 m with clay, silt clay and sand clay loam texture (Mohamed et al. 2019).

In common with many industrialising regions, environmental pollution by PTE is a net risk to soil function, impacting on agriculture as well as settlements in and around the area. Due to the installation of the Aswan Dam in the 1960s, the sediment load for downstream input is compromised and naturally fertile soils on the Nile banks have had an increasingly restricted input of natural nutrients. Consequently, fertiliser use is high and inputs to soils from these sources, and increased pesticide use and high industrial inputs have increased stress on this sensitive ecosystem (Badawy et al. 2017). Soil contamination with PTE has previously been identified as an issue in parts of the Helwan area ((Shakour 1991); (Ammar 2005); (Melegy 2005); (El-Mekawy 2007); (Melegy et al. 2010) and (Alkhdhairi et al. 2018)). These have already been associated with crop production in this area ((El-Mekawy 2007); (Galal et al. 2016) and (Alkhdhairi et al. 2018)) as a result of the presence of high PTE concentrations (particularly for Zn, Pb, Cu and Cd) in vegetation in the study area. The identification of wide spread soil contamination in the El-Tabbin district of southern Helwan (Melegy et al. 2010) highlighted Cd, Se, As, Zn, Ba and Pb as an issue.

El Tebbin region presents an opportunity to study the interaction of the cumulative industrial emissions on a mixed land use area. With the importance of residential and agricultural community and poorly developed agricultural soils, there is considerable interest in developing the database of soil status. Despite the considerable history of human habitation in the region, this is relatively poorly documented and understanding the reactivity and availability of soil-based pollution sources is critical for future environmental management in the area and in zoning locations for detailed strategic development. We focus on identifying factors controlling PTE distribution and availability in El Tebbin soil and estimating their mobility and bioavailability.

## Materials and methods

### Soil sampling and chemical analysis

Soil samples were collected from locations across the region during field campaigns in 2012. A total of 32 locations across different land use types were identified for soil sample collection in the Helwan area (Fig. 1 and Table 1) and hand dug from 0 to 10 cm, taken to the laboratory before air drying (if needed) and sieving to < 2 mm. Sampling site locations were recorded by a Global Positioning System (Garmin GPS v) and sampling map generated using GIS software (Desktop 2014). Soil samples were examined for their pseudo-total PTE concentration of seven trace elements (Co, Zn, Cd, Ni, Pb, Cu and Cr) and a number of physiochemical parameters. Total PTE were determined by digestion of with aqua regia (3 HCl: 1 HNO_3_) and analysed using atomic absorption spectrophotometer (Buck scientific 205AA, Norwalk, USA). Quality assurance and control was assessed through replicate analysis and selection of most appropriate absorption wavelength. Calibration standards were matrix matched and blank control samples run periodically. Soil pH was measured in 1:1 soils to water ratio by using HANNA (HI93300) combined electrode (Hanna Instruments srl, Italy). Calcium carbonate percentage (CaCO_3_%) was estimated by the titrimetric methods. Soil organic matter percentage (SOM %) was determined according to the modified Walkley and Black method (USDA 2014). High purity chemicals (AR grade), double-distilled water, disposable plastics, clean apparatus and glassware were used during all stages of samples collecting, handling and analysing to prevent contamination. All measurements were completed in triplicate for precision.

**Table 1:** Summary of geographically dispersed activities in the El Tebbin area, identified at the study sampling sites in Fig. 1, which may act as diffuse or direct sources for PTEs to soil profiles

Sample no.	Activity (nearby source/activity)
1	Urban
2	Somid Company of pipes
3	North El Marazeeq Bridge
4	Egyptian Electric Holding Company
5	Coke Plant
6	El Tebbin Thermel Power Plant
7	Road
8	Road
9	Road
10	Road
11	Road
12	Road
13	Road
14	Industrial Complexes
15	Cement Factory and road
16	El Hager drain
17	Iron and steel company and road
18	Road
19	Road
20	Road
21	Road
22	Industrial complex
23	Industrial complex and road
24	Industrial complex and road
25	Coke Plant
26	Rural
27	Rural
28	Rural
29	Rural
30	Rural
31	Rural
32	Rural

### Contamination factor

Contamination level was assessed using the contamination factor (CF) recognised in (Hakanson 1980) based on the following equation:-

1 $$ \mathrm{CF}=\mathrm{Cs}/\mathrm{Cb} $$

Where Cs is the concentration of metal in the study samples and Cb is reference value. Baseline concentration of (Turekian and Wedepohl 1961) and mean World soil (Kabata-Pendias 2010) were used as Cb in this study (Mn = 850 mg/kg, Co = 19 mg/kg, Ni = 68 mg/kg and Pb = 20 mg/kg, Cd = 0.5 mg/kg, Cu = 20 mg/kg and Cr = 90 mg/kg). Levels of contamination have previously been classified (Hakanson 1980) based on the contamination factor value as the following; CF < 1 low; 1 < CF < 3 moderate; 3 < CF < 6 considerable and CF > 6 as high contamination.

### Sequential chemical extraction

Sequential chemical extraction of PTE in the five chemical fractions was carried out using the 5 step procedure outlined above (Tessier et al. 1979) which was initially developed for use in soil and sediment characterisation. Experimental details including sample:solution ratios, contact time and laboratory conditions were as detailed in Tessier et al. 1979. Aliquots of 2 g samples were extracted in duplicate and extracts analysed as above for target elements. Success of extraction was assessed using Eq. 2 below:

2 $$ \mathrm{Recovery}\%=\left[\mathrm{F}1+\mathrm{F}2+\mathrm{F}3+\mathrm{F}4+\mathrm{F}5\right]/\left[{\mathrm{Ctotal}}^{\ast}\;100\;\right] $$

The recoveries of Co, Ni, Zn, Cd, Pb and Cu in each extraction procedure ranged from 96.3 to 100.2%, 100.0 to 100.3%, 100.1 to 100.1%, 100.0 to 103.7%, 100.0 to 100.1% and 100.0 to 100.3%, respectively. The potential mobility (MF) of six trace elements in soils (Co, Zn, Cd, Ni, Pb and Cu) was also derived using equation no. 3 described by (Kabala and Singh 2001).

3 $$ \mathrm{MF}={\left[\mathrm{F}1+\mathrm{F}2+\mathrm{F}3+\mathrm{F}4/\mathrm{F}1+\mathrm{F}2+\mathrm{F}3+\mathrm{F}4+\mathrm{F}5\right]}^{\ast }100 $$

Bioavailability was estimated in terms of risk assessment code (RAC) calculated from (Jain 2004) using the following two equations (eq. 4 and 5) for neutral-alkaline and acidic soil respectively:

For neutral to alkaline soil (Non-acidic soil)

4 $$ \mathrm{RAC}=\mathrm{F}1\%/\mathrm{total} $$

For acidic soil.

5 $$ \mathrm{RAC}=\left(\mathrm{F}1+\mathrm{F}2\right)\%/\mathrm{total} $$

### Statistical analyses

Both descriptive and multivariate statistical analyses were used to interpret the data using SPSS 16.0 software.

## Result and discussion

### PTE content and soil characteristics

A summary of data (summary analytical statistics) is provided in Table 2. Comparing our data with previous studies (Table 3) indicates that the Helwan is more contaminated with Cd and Pb than the opposite (west) bank of the River Nile (Giza area), which we attribute to the dominance of heavy industries in Helwan compared to Giza. The contamination factor (CF) identified clustering of elements within the group of Co, Zn, Cd, Ni, Pb, Cu and Cr (Table 4). Based on CF values, the study area showed a range from low to very high contamination levels, with Co strongly enriched across 75% of the studied sites (CF > 6). The entire region showed high Zn contamination (CF > 6), while Cd was enriched in a smaller number of locations (samples 16, 22, 24 and 25 with CF ≥ 6). Soil ranged from low to moderate contaminated with respect to Ni and Cu, while from low to very high contamination with Pb. The Cr data were close to background in all sites, and values for Cr were within limits for unpolluted agricultural soils (CF < 1). The Cr content of soils exhibits wide variation among different Egyptian areas (Table 3). Abdel-Sabour et al. 1998 has attributed this variation in Cr content to soil types and land management. However, Cr content in this study is in line with that found by Abdel-Sabour and Zohny 2004 (17.57 to 25.0 ppm) in unpolluted alluvial soil of Nile Delta and within the range of the average of Cr content of different Egyptian soil (11.6 to 179 ppm) reported by Abdel-Sabour et al. 1998.

**Table 2:** Summary descriptive statistical analysis of soil data from samples from El Tebbin, Egypt

Parameter		EC	CaCO_3_	SOM	Co	Zn	Cd	Ni	Pb	Cu	Cr	Clay	Silt	Sand
		(μS/cm)	(mg/kg)	(mg/kg)	(mg/kg)	(mg/kg)	(mg/kg)	(mg/kg)	(mg/kg)	(mg/kg)	(mg/kg)	(%)	(%)	(%)
Mean		6005.5	17.4	4.3	286.8	210.4	0.9	77.1	75.2	42.0	32.6	15.1	37.6	46.6
Std. error of mean		1411.7	2.2	0.3	33.3	96.7	0.4	6.1	18.7	11.8	1.1	1.2	2.3	2.4
Median		1753.0	14.6	4.0	340.7	57.7	0.0	83.8	50.3	33.1	33.5	15.6	38.7	50.3
Std. deviation		7985.7	12.6	1.8	188.6	547.1	2.5	34.7	105.9	67.0	6.2	6.6	13.3	13.5
Skewness		1.5	0.8	0.8	− 0.5	4.7	4.1	− 0.1	2.8	5.4	− 0.2	0.2	− 0.1	− 0.4
Kurtosis		0.9	− 0.3	0.7	− 1.0	23.3	18.9	− 0.6	7.3	30.1	− 0.9	− 0.5	0.2	− 0.6
Minimum		135.0	2.1	1.5	6.5	27.0	0.0	8.5	4.0	14.0	21.0	2.1	4.1	15.8
Maximum		27,265.0	46.5	9.4	611.3	3000.0	13.0	143.9	432.1	404.0	44.0	27.6	64.2	70.6
Percentiles	25	868.5	6.5	3.0	15.8	36.0	0.0	46.2	15.0	19.5	27.3	9.8	29.0	36.3
	50	1753.0	14.6	4.0	340.7	57.7	0.0	83.8	50.3	33.1	33.5	15.6	38.7	50.3
	75	7513.8	24.4	5.5	418.8	111.9	0.2	102.4	72.1	38.7	37.8	19.5	45.6	58.2
CV%		133.0	72.6	42.0	65.8	260.0	280.4	45.0	140.8	159.6	19.2	43.8	35.4	28.9

**Table 3:** Summary of soil PTE content from locations in Egypt in comparison to results of this study

Parameter	Co	Zn	Cd	Ni	Pb	Cu	Cr
	(mg/kg)	(mg/kg)	(mg/kg)	(mg/kg)	(mg/kg)	(mg/kg)	(mg/kg)
Current study	6.5–611.3	27–3000	0–13	8.5–143.9	4–432.1	14–404	21–44
Aswan agricultural soil (Darwish and Pöllmann 2015)	16.6–54.9	628–2224	8.3–28.3	23.1–98.4	15.9–42.7	20.2–77.5	60–218.2
Middle Egypt (Asyut to Cairo) (Badawy et al. 2017)	5–36	13–165	–	8–84	–	–	33–308
El-Mahla El-Kobra Area (Mahmoud and Ghoneim 2016)	–	54–449	11–33	31–164	48–92	60–386	–
Giza area (Salman et al. 2018)	–	–	1.25–2.1	–	42.1–196.1	–	141.7–297.6

**Table 4:** Soil contamination factors for PTEs calculated for locations samples in this study (range and mean)

Contamination factor (CF)	Co	Zn	Cd	Ni	Pb	Cu	Cr
Mean	15.1	2.2	1.8	1.1	3.8	2.1	0.4
Minimum	0.3	0.3	0.0	0.1	0.2	0.7	0.2
Maximum	32.2	31.6	26.0	2.1	21.6	20.2	0.5

The contamination factor (CF) integrated with descriptive analysis was used to assess the anthropogenic influence on PTEs. The CF approach used reference levels which were in line with more recent assessment (Badawy et al. 2017), and coupled with the variability, the identification of disrupted locations seems a reasonable result. A significant variability in the soil Pb, Zn, Cd, Cu content (C.V = 140.8%, 260.0%, 280.4% and 159.6% respectively) highlights likely anthropogenic disturbance in addition to natural inputs. The more uniform distribution of Cr (the lowest C. V (19.2%) and low CF suggest a strong natural background origin. Such conclusion is linked to results commonly reported in the literature (Omer 1996) and (Salman 2013) that characterise Cr as a geogenic element in the region. This attributed to the nature of parent material in the hinterlands especially the basaltic rocks in the Ethiopian plateau and the sedimentary depositional environments, despite limitation of sediment transport through the Aswan Dam. Although Co exhibited very high contamination factors, the distribution pattern of Co shows generally low/moderate variability (C.V = 65%). This suggests anthropogenic supply of Co is limited. The elevated Co may be attributed to the nature of parent material in the basaltic rocks of the Ethiopian plateau (Omer 1996) and (Salman 2013), or more diffuse sources even agricultural inputs through animal feed. Data for Ni ranged from low to moderate contaminating level (Table 4) and with its low coefficient of variation (CV = 45.0%) also suggests its natural origin and the limited occasional influence of anthropogenic inputs. The texture of the studied soil was found to be silty sand, sandy mud, sandy silt and muddy sand. Clay content ranged from 2.1 to 27.6%, silt from 4.1 to 64.2% and sand from 15.8 to 70.6%. The pH range varied from 6.57 to 8.51. At the moderately low pH, the relatively bioavailable forms of PTEs are expected to be favoured. Their solubility is generally greater in normal agriculture soils with pH ranged from 5.0 to 7.0 (Sanders 1983) and (Alloway 1990). The total CaCO_3_ content ranges between 2.1 and 46.5% *w*/*w*. Organic matter ranged from 1.9 to 9.4% *w*/*w*. Generally, the areas adjacent to the road, such as (8, 10, 11, 15 and 19), experience the highest organic matter content coupled with considerable contamination by lead compared to those further away. This suggests fuel combustion residues as the main source responsible for the excess SOM content.

### PTE fractionation in soil

**Fig. 2:**
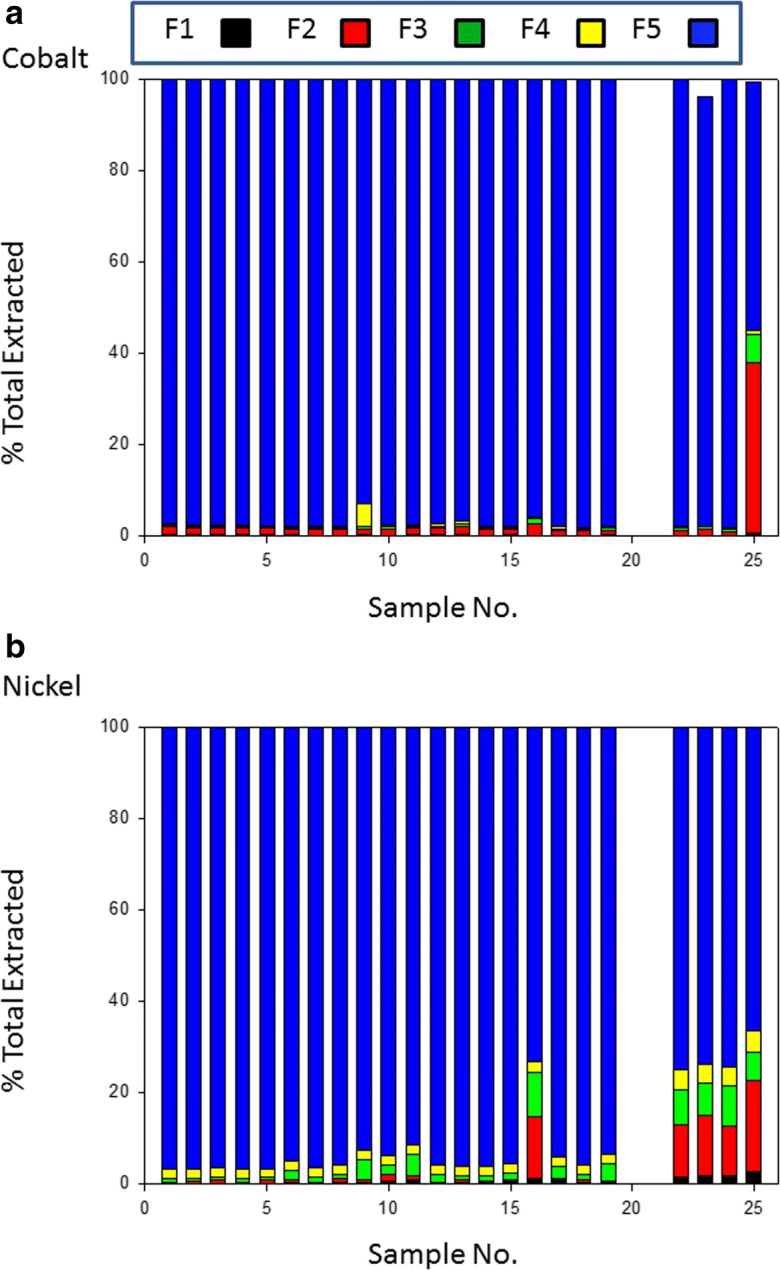

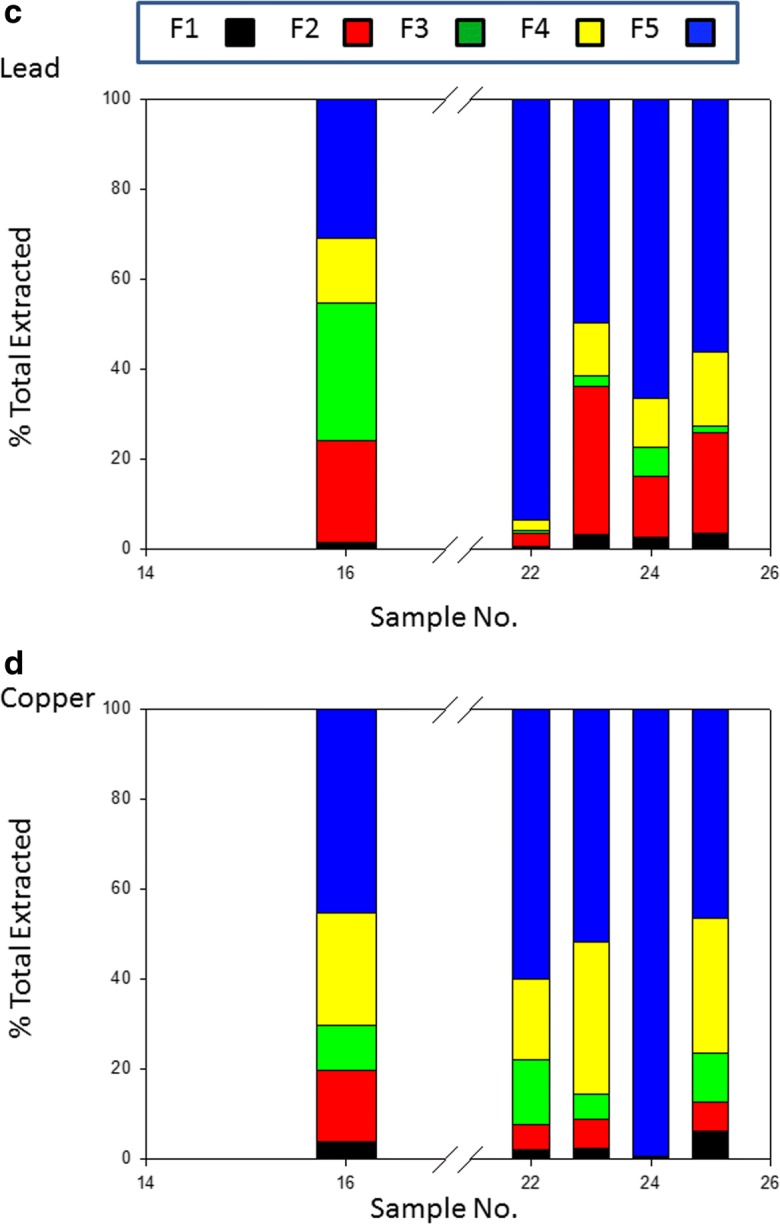

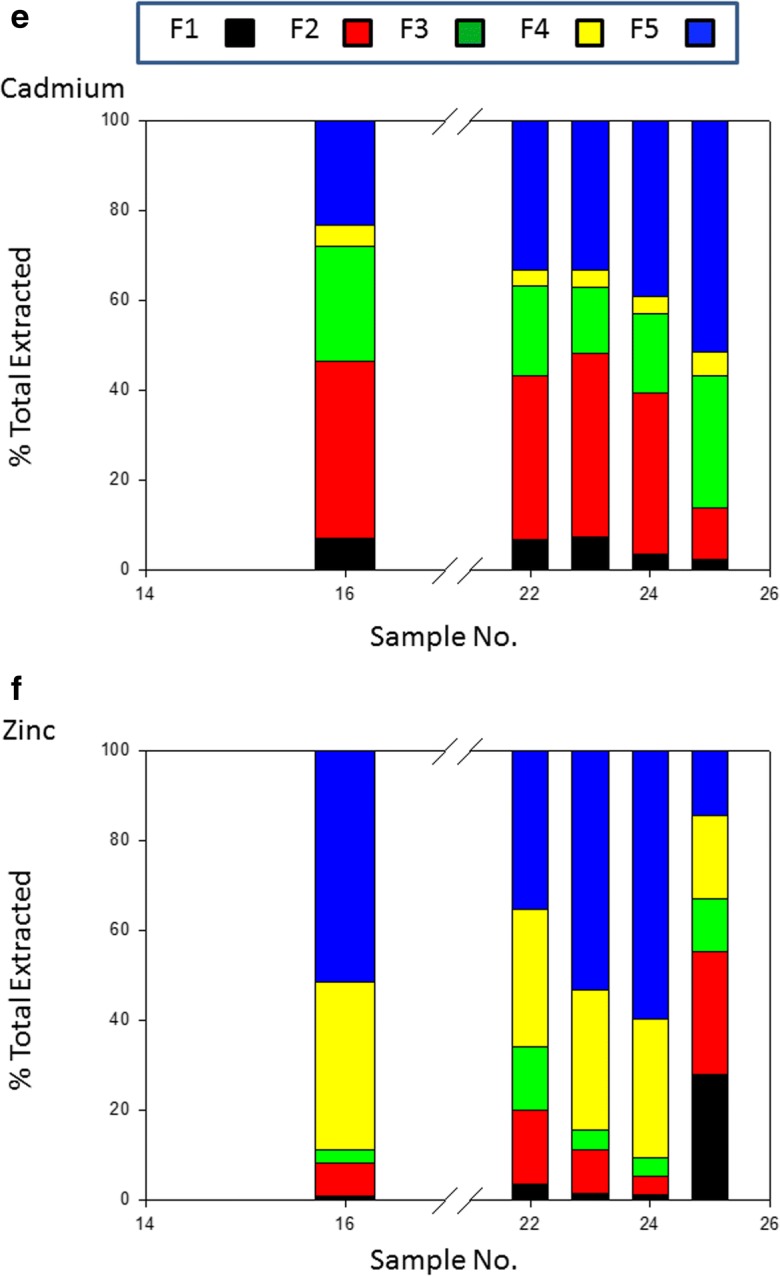
Percentage of total metal associated with sequential extraction phases. F1 exchangeable, F2 carbonate, F3 reduced, F4 oxidised, F5 residual (see text for details). (a) cobalt, (b) nickel, (c) lead, (d) copper, (e) cadmium and (f) zinc

A subset of the samples found to be the most highly contaminated by Co, Ni, Pb, Cu, Cd and Zn were selected for sequential extraction. The PTE distribution among the five extracts is shown in Fig. 2(a–f). The more available fractions of Co and Ni are extremely depleted in the soil (mean = 4.4% and 9.5%), with a minor exception observed in sample no. 25 (45.4% and 33.4% respectively) (Fig. 2(a, b)) which is close to potentially disruptive anthropogenic activities. Site 25 is of special interest as it is downwind from a coke plant and is also used for agriculture (Fig. 1 and Table 1). It has been estimated (Shakour 1991) that the highest annual mean concentration of suspended particulate in air near the coke plant in Helwan industrial area is in the region of 3500 μg/m^3^. The Co and Ni content is partitioned predominantly in resistant fraction averaging 95.6% and 90.5% respectively. This supports the geogenic origin of Co and Ni with very limited anthropogenic inputs statistically identified in the previous section. The more mobile fractions of Pb and Cu average 40.7% and 39.2% of total respectively, while the non-residual fractions of Cd and Zn are higher than residual one, averaging, 62.6% and 57% respectively. Enrichment of the PTEs (Cd, Zn, Pb and Cu) in bioavailable fractions reflects the likely intensity of anthropogenic influence.

The average concentrations of the PTEs in the different fractions in descending order were as follows:

- Co: Residual > Carbonate > Reducible > Organic > Exchangeable
- Ni: Residual > Carbonate > Organic > Reducible > Exchangeable
- Pb: Residual > Carbonate > Reducible > Organic > Exchangeable
- Cu: Residual > Reducible > Organic > Carbonate > Exchangeable
- Cd: Residual ≥ Carbonate = Organic > Exchangeable = Reducible
- Zn: Carbonate > Residual ≈ Reducible > Exchangeable > Organic

The highest residual fraction was observed for Co and Ni at 95.6% and 90.5% respectively, confirming a strong lithogenic influence. The significant portion of Pb in carbonate fraction contributed to the known affinity of Pb toward carbonate (co-precipitation with carbonate minerals) and may be a feature of both natural parent material and the influence of carbonate on metal mobility effectively immobilising Pb by providing an adsorbing or nucleating surface and by buffering pH). Significant amounts of Pb in the organic fraction of the soils also suggested that Pb may have high formation constants of organic-Pb complexes. Among non-residual fractions, the FeMnOX fraction hosted the highest percentages of the total Cu. This may be attributed to the high stability of FeMnOX -Cu compounds. Iron and manganese-oxides have high scavenging efficiencies for trace elements from solution through processes such as adsorption and co-precipitation (Sundaray et al. 2011). This result in agreement with results elsewhere which have reported the dominance of Cu in Fe-Mn oxides (Zhao et al. 2006) and (Shaheen et al. 2013). The strong association of Cd with the carbonate fraction is most probably due to the similarity of the ionic radii of Cd (0.97 A) and Ca (0.99 A). In neutral and alkaline soils and also under oxidation conditions, Cd is likely to form CdCO_3_ (Kabata-Pendias 2010). The presence of carbonate minerals effectively immobilises Cd. The highest percentage of Zn was found in carbonate fraction of the soils sampled. In soils of Iran and Zambia showing a relatively high Zn bearing carbonate fraction, Saffari et al. 2009 and Chirwa and Yerokun 2012 reported similar results compared to these El Tebbin soils. Generally, CaCO_3_ tends to adsorb Zn or form complex solid phases such as CaCO_3_: ZnCO_3_ at high pH values (Ramos et al. 1999). In addition, a significant portion of Zn was found in residual and Fe-MnO fractions. This feature has been reported by Dvorak et al. 2003, Milivojević et al. 2005, and Saffari et al. 2009, where stabilisation of Zn in the Fe-MnO fraction occurs in soil.

### PTE mobility and bioavailability

**Fig. 3:**
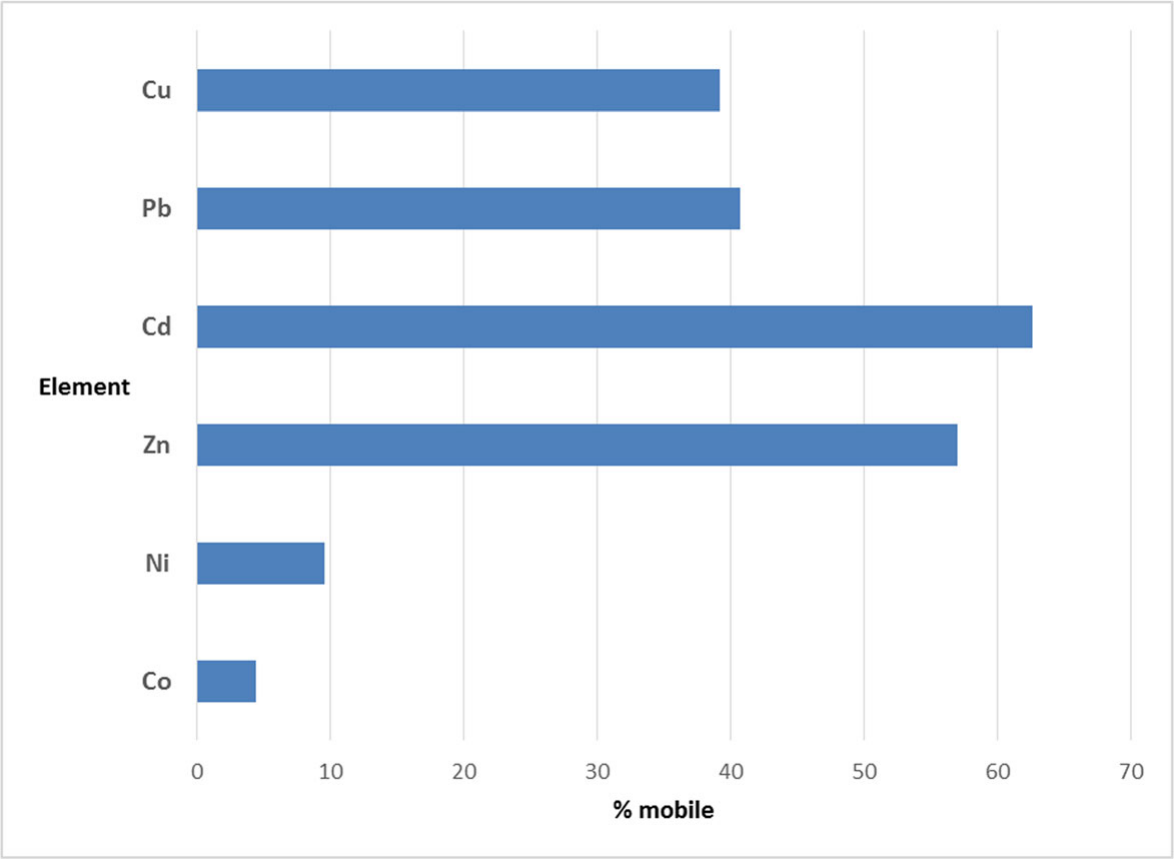
Mean potential mobility (sum of F1–4 as a % of total) for different metals in El Tebbin soil samples

The mobility of elements in the soil could be assessed based on relative content of fractions weakly bound to the soil constituents. The relative index of elements mobility was calculated as a mobility factor (Kabala and Singh 2001). The mobility factor (MF) values were graphically represented for each element (Fig. 3) and potential mobility decreases in the order: Cd > Zn > Pb > Cu > Ni > Co. The relatively high potential mobility for Zn and Cd (> 50%) may be due to a higher anthropogenic contribution and solubility of common solid phases formed during mobilisation. Although the residual fraction is important for the PTEs studied, the significant portion in the potentially mobile fractions indicates that they still may pose some environmental risk. These portions of Pb, Cu, Cd and Zn under favourable conditions are available for wider food chain impact through plant uptake. Elevated concentrations of Cd, Zn, Pb and Cu recorded in some plant species collected from the study area by El-Mekawy 2007, Galal et al. 2016 and Alkhdhairi et al. 2018 confirm the bio-accessibility and total bioavailability indicated by this result. The content of the exchangeable fraction (F1) is readily available for plant uptake. Element mobility in F2 increases with lowering pH and carbonate fractions become more sensitive to degradation and release. Oxidising conditions expose the elements associated with the organic fraction (F3) while reducing conditions release trace elements bonding with Fe-MnO fraction. The seasonal tillage and high temperature will lead to the degradation of organic matter, and hence liberation of metals. Also, agrochemicals and urban and industrial surface water runoff may also affect the soil pH.

From our findings, the average bioavailability of PTEs declines in order as Zn > Cd > Cu > Pb > Ni > Co. Correspondingly, the general trends of the relative abundance of trace elements in vegetation studied previously in the area (El-Mekawy 2007) and (Alkhdhairi et al. 2018), were Cd > Cu > Pb > Cr > Co and Zn > Pb > Cd > Cu respectively, supporting these findings.

### Multivariate statistical analysis

#### Principle components analysis

Four principal components were extracted from the investigated soil data explaining 76.1% of the variance (Table 5). The first component (PC1) is responsible for 25.4% of the total variance and shows significant positive loadings for Cr, clay, and silt all inversely correlated with carbonate and sand. The Cr is chiefly controlled by the natural composition of soil. It was very close to background value and its coefficient of variation is very low suggesting natural factor controlling its distribution. Hence, PC1 can be named as “geogenic component”. The second component (PC2) explains 23.4% the variance in the dataset. PC2 includes pH, bioavailable fraction of Ni, Pb, Zn and Cu. Positive correlation between available fraction of Ni mostly contributed from anthropogenic sources and total concentration of Pb, Zn and Cu suggest anthropogenic origin. The input of Pb is known to be controlled by anthropogenic activity connected with traffic and industrial processing. This confirms the anthropogenic origin of PC2. Although the content of Cu exhibited a relatively low contamination level, its high coefficient of variation and strong correlation with anthropogenic Pb, as well as bioavailable Ni, suggest a partial anthropogenic component. Increasing pH decreases Pb, Zn and Cu solubility, which is the usual trend of cationic metals, retaining them in the soil. In contrast, the solubility of Ni increases with increasing pH, as with all anionic species, removing them from the soil decreasing the total Ni content. That is why total Ni concentration is inversely related to pH in this factor. The effect of pH on element distribution in the soils is significant, and PC2 can be denoted as “pH factor” underlining the influence of soil processes on PTE distribution. The PC3 accounts for over 16% of the total variance. It has a high loading for (clay, Pb as well as bioavailable fractions of Co and Ni). However, the lowest anthropogenic contribution is suggested for Co and Ni but anthropogenic sources cannot be completely excluded. Bioavailable fractions are strongly influenced by anthropogenic sources. As Pb also is known as an anthropogenic marker element, particularly in a region with low natural background, a positive correlation between bioavailable fractions, Pb and clay could be suggested PC3 as a second anthropogenic component that is texturally dependent. The final PC4 includes Cu, Ni and Zn and may represent a second “geogenic factor”. Enrichment of Ni in the residual fraction and low contamination level of Cu confirm their natural origin. Distribution of these elements in more than one factor confirmed their mixed origin previously proved from descriptive statistical and sequential extraction. The soil element content shows a mix of parent material from which soil is formed, soil processes - pH, texture and anthropogenic inputs. These conclusions fit with studies elsewhere on source identification in urban/rural soils (Rodrigues et al. 2009).

**Table 5:** Summary of principal component analysis of soil data from El Tebbin sites: loadings of including variance % and cumulative %

Variable	Component			
	PC1	PC2	PC3	PC4
CaCO3	*− .613*	− .001	.087	− .240
Co	− .309	*.640*	.076	.103
Zn	.122	*.859*	− .132	*.334*
Ni	− .332	*− .570*	.005	*.590*
Pb	− .222	*.556*	*.639*	.174
Cu	.000	*.273*	.104	*.869*
Cr	*.840*	.005	.033	− .147
Clay%	*.653*	.053	*.614*	.077
Silt%	*.800*	− .086	− .175	− .051
Sand%	*− .909*	.009	− .101	.226
pH	− .080	*.758*	.394	− .096
Ni*	.199	*.767*	*.485*	− .048
Co*	− .077	.132	*.939*	.018
Variance%	25.4	23.4	16.5	10.8
Cumulative%	25.4	48.8	65.3	76.1

Values in *italics* show the most significant contributions to variability

Extraction method: principal component analysis. Rotation method: Varimax with Kaiser-Meyer-Olkin normalisation (KMO = 0.598). a. Rotation converged in 6 iterations. Ni*, bioavailable fraction; Co*, bioavailable fraction. Total variance 76.1%

#### Cluster analysis and spatial associations

**Fig. 4:**
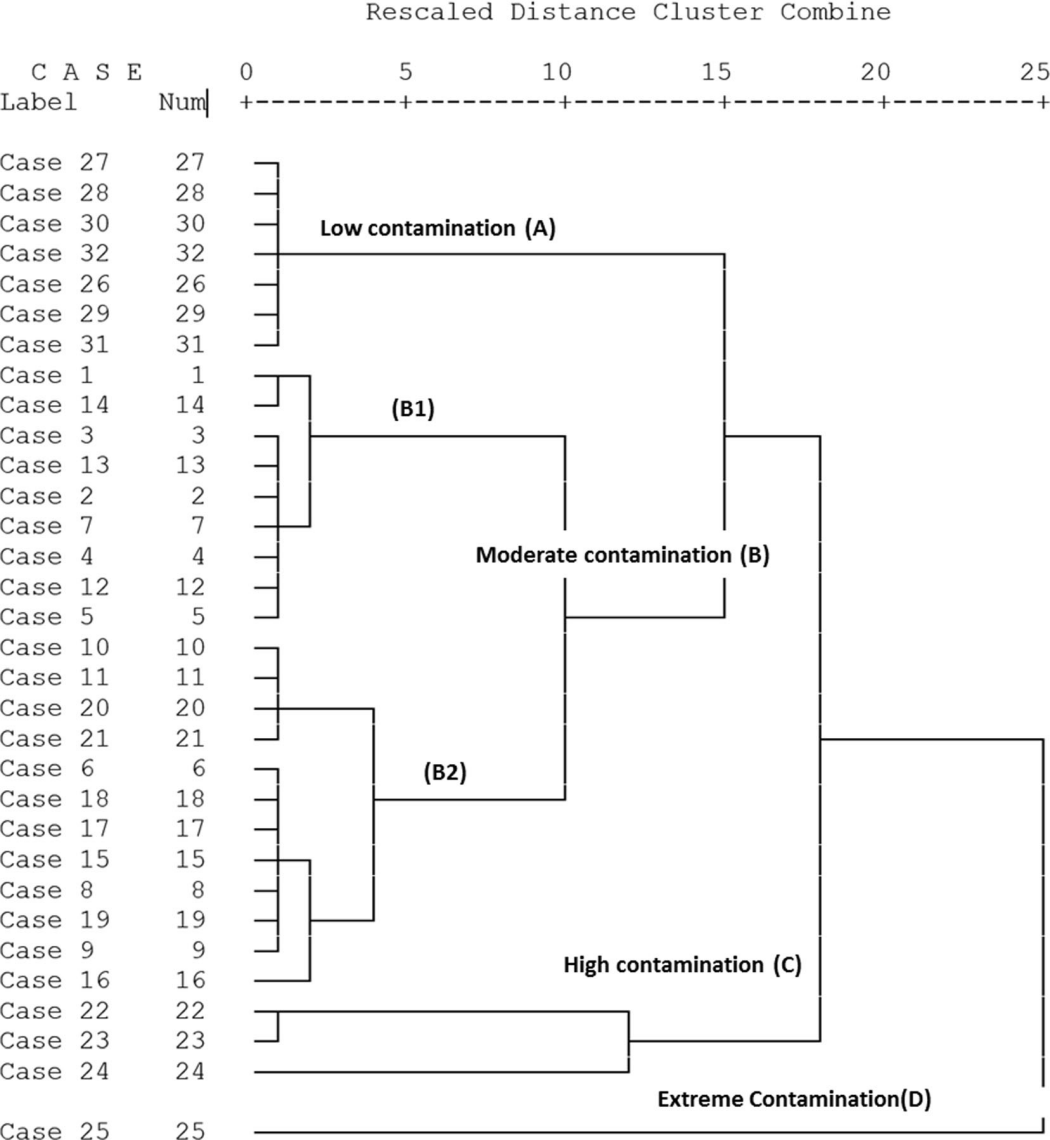
Hierarchical cluster analysis using Ward Method for calculated contamination factors of the soils studied

Cluster analysis classified the samples into four groups according to level of PTE contamination (Fig. 4), with Co was omitted as discussed above. Cluster (A) represents the uncontaminated sites with respect to the elements studies (CF < 1). This may be attributed to their sample location being most remote from and up wind of major industrial point sources, in rural zones. However, this group is moderately contaminated with Cu probably as result of agrochemical application. As reported, Cu is usually considered as a marker element of agricultural activities (Acosta et al. 2011) and is specifically related to application of commercial fertilisers (including both inorganic and organic animal wastes) and pesticides, e.g. measured Cu 7.5 μg kg in local fertilisers (Salman et al. 2017). Generally cluster (B) includes low to moderately contaminated sites, with a few exceptions (subgroup B2) which are characterised by Pb contamination and close vicinity to the road network, and therefore more directly affected by traffic emissions. It has been estimated (El-Mekawy 2007) that the monthly mean concentration of Pb in dust fallout in the study area is in the region of 1420 μg m^2^. Sample no. 16 from subgroup (B2) showed a high contamination factor for Cd (CF = 9), and can be seen as an outlier, furthest from the other subgroup samples (Fig. 4). The excess of Cd is perhaps due to influence of contaminated waste water from nearby drain system (El Hager drain). Groups C and D include the highest contamination sites (CF > > 6) with the 4 PTE Co, Zn, Pb and Cd. These are the sites closest to the industrial complexes (coke and iron and steel plants), and traffic emissions from nearby roads. Site 25 is of special concern as it showed the highest level of pollution from Zn and Cd, and is localised downwind from the coke plant. Hence, it existed only as isolated sample, which can be considered as outlier due to the anomalous excess of Zn and Cd. In addition, group C generally has higher Cd and Zn contamination.

## Conclusion

Spatial variability of trace elements distribution (Co, Ni, Cr, Cd, Zn and Pb) in El Tebbin soil suggests the behaviour of them is controlled by more than one factor. The multivariate analysis highlights the relative control on PTE content from the potential anthropogenic verses geogenic sources. PTE bioavailability varies greatly and shows different influences spatially across the sampling areas. Higher portions of bioavailable fractions are mostly determined by anthropogenic inputs. A number of the elements studied (Cd, Zn, Cu and Pb) show significant portion of soil metal content in non-resistant fractions which under changing conditions (e.g. pH) may be mobilised. This is of significance for the bioaccumulation highlighted previously. This study provides evidence to support the influence of bioavailability for further food chain transfer. Traffic flow, discrete industrial activities and the application of agrochemicals appear to contribute varying degrees of input to the soils and from cluster analysis, supported by PCA, the locations are identifiable. The high concentration of PTE in roadsides soils and the soils close to coke factory is a matter of concern, particularly for changes to soil conditions as the parent materials contain significant carbonate components which act as source of elements and also may help inhibit further mobility. The sensitivity of these materials to urbanisation or changes in land use is likely to release further into the food chain, given the demand for productive agricultural land by the local population. We have used geochemical tools to identify soil sensitivity and further areas for evaluation and recognise the competing inputs from sources close to industrial and urban developments.
